# Simple Stochastic Games with Almost-Sure Energy-Parity Objectives are in NP and coNP

**DOI:** 10.1007/978-3-030-71995-1_22

**Published:** 2021-03-23

**Authors:** Richard Mayr, Sven Schewe, Patrick Totzke, Dominik Wojtczak

**Affiliations:** 8 University of Oxford, Oxford, UK; 9 Sorbonne Université - LIP6, Paris, France; 10 University of Edinburgh, Edinburgh, UK; 11 University of Liverpool, Liverpool, UK

**Keywords:** Simple Stochastic Games, Parity Games, Energy Games

## Abstract

We study stochastic games with energy-parity objectives, which combine quantitative rewards with a qualitative $\omega$-regular condition: The maximizer aims to avoid running out of energy while simultaneously satisfying a parity condition. We show that the corresponding almost-sure problem, i.e., checking whether there exists a maximizer strategy that achieves the energy-parity objective with probability 1 when starting at a given energy level *k*, is decidable and in $\mathsf {NP}\cap \mathsf {coNP}$. The same holds for checking if such a *k* exists and if a given *k* is minimal.

### 1 Introduction

*Simple stochastic games* (SSGs), also called *competitive Markov decision processes* [30], or $2\frac{1}{2}$-*player games* [23,22] are turn-based games of perfect information played on finite graphs. Each state is either random or belongs to one of the players (maximizer or minimizer). A game is played successively moving a pebble along the game graph, where the next state is chosen by the player who owns the current one or, in the case of random states, according to a predefined distribution. This way, an infinite run is produced. The maximizer tries to achieve an objective (in our case almost surely), while the minimizer tries to prevent this. The maximizer can be seen as a controller trying to ensure an objective in the face of both known random failure modes (encoded by the random states) and an unknown or hostile environment (encoded by the minimizer player).

Stochastic games were first introduced in Shapley's seminal work [46] in 1953 and have since then played a central role in the solution of many problems in computer science, including synthesis of reactive systems [45,42]; checking interface compatibility [27]; well-formedness of specifications [28]; verification of open systems [4]; and many others.

A huge variety of objectives for such games was already studied in the literature. We will mainly focus on three of them in this paper: parity; mean-payoff; and energy objectives. In order to define them we assume that numeric rewards are assigned to transitions, and priorities (encoded by bounded non-negative numbers) are assigned to states.

The *parity objective* simply asks that the minimal priority that appears infinitely often in a run is even. Such a condition is a canonical way to define desired behaviors of systems, such as safety, liveness, fairness, etc.; it subsumes all $\omega$-regular objectives. The algorithmic problem of deciding the winner in non-stochastic parity games is polynomial-time equivalent to the model checking of the modal $\mu$-calculus [49] and is at the center of the algorithmic solutions to the Church's synthesis problem [44]. But the impact of parity games goes well beyond automata theory and logic: They facilitated the solution of two long-standing open problems in stochastic planning [29] and in linear programming [32], which was done by careful adaptation of the parity game examples on which the strategy improvement algorithm [31] requires exponentially many iterations.

The parity objective can be seen as a special case of the *mean-payoff objective* that asks for the limit average reward per transition along the run to be non-negative. Mean-payoff objectives are among the first objectives studied for stochastic games and go back to a 1957 paper by Gillette [33]. They allow for reasoning about the efficiency of a system, e.g., how fast it operates once optimally controlled.

The *energy objective* [14] can be seen as a refinement of the mean-payoff objective. It asks for the accumulated reward at any point of a run not to be lower than some finite threshold. As the name suggests, it is useful when reasoning about systems with a finite initial energy level that should never become depleted. Note that the accumulated reward is not bounded a-priori, which essentially turns a finite-state game into an infinitely-state one.

In this paper we consider SSGs with *energy-parity* objectives, which requires runs to satisfy both an energy and a parity objective. It is natural to consider such an objective for systems that should not only be correct, but also energy efficient. For instance, consider a robot maintaining a nuclear power plant. We not only require the robot to correctly react to all possible chains of events (parity objective for functional correctness), but also never to run out of energy as charging it manually would be risky (energy objective).

While the complexity of games with single objectives is often in $\mathsf{NP} \cap \mathsf{coNP}$, asking for multiple objectives often makes solving games harder. Parity games are commonly viewed as the simplest of these objectives, and some traditional solutions for non-stochastic games go through simple reductions to mean-payoff or energy conditions (which are quite similar in non-stochastic games) to discounted payoff games that establishes the membership of those problems in UP and coUP [35]. However, asking for *two* parity objectives to be satisfied at the same time leads to $\mathsf{coNP}$ completeness [21].

We study the almost sure satisfaction of the energy-parity objective, i.e., with probability 1. Such *qualitative analysis* is important as there are many applications where we need to know whether the correct behavior arises almost-surely, e.g., in the analysis of randomized distributed algorithms (see, e.g, [43,47]) and safety-critical examples like the one from above. Moreover, the algorithms for *quantitative analysis*, i.e., computing the optimal probability of satisfaction, typically start by performing the qualitative analysis first and then solving a game with a simpler objective (see, e.g., [23,15]). Finally, there are stochastic models for which qualitative analysis is decidable but quantitative one is not (e.g., probabilistic finite automata [6]). This may also be the case for our model.

**Our contributions.** We consider stochastic games with energy-parity winning conditions and show that deciding whether maximizer can win almost-surely for a given initial energy level $k$ is in $\mathsf{NP} \cap \mathsf{coNP}$. We show the same for checking if such $k$ exists at all and checking if a given $k$ is the smallest possible for which this holds. The proofs are considerably harder than the corresponding result for MDPs [40] (on which they are partly based), because the attainable mean-payoff value is no longer a valid criterion in the analysis (via combinations of sub-objectives). E.g., even though the stored energy might be inexorably drifting towards $+\infty$ (resp. $-\infty$), the mean-payoff value might still be zero because the minimizer (resp. maximizer) can delay payoffs for longer and longer (though not indefinitely, due to the parity condition). Moreover, the minimizer might be able to choose between different ways of losing and never commit to any particular way after any finite prefix of the play (see Example 1).

Our proof characterizes almost-sure energy-parity via a recursive combination of complex sub-objectives called *Gain* and *Bailout*, which can each eventually be solved in $\mathsf{NP} \cap \mathsf{coNP}$.

Our proof of the $\mathsf{coNP}$ membership is based on a result on the strategy complexity of a natural class of objectives, which is of independent interest. We show (cf. Theorem 6; based on previous work in [34]) that, if an objective $\mathcal{O}$ is such that its complement is both shift-invariant and submixing, and that every MDP admits optimal finite-memory deterministic maximizer strategies for $\mathcal{O}$, then the same is true in turn-based stochastic games.

*Example 1.* Fig. 1 shows an energy-parity game that the maximizer can win almost surely when starting with an energy level of $\geq 2$ from the middle left node. Whenever the game is at that node with an energy level $\geq 3$, then the maximizer can turn left and has at least $\frac{1}{2}$ chance that the energy level will never drop to 2 while wining the game with priority 2. This is because we can

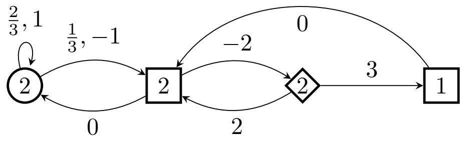

Fig. 1: A SSG with two maximizer states (□), one minimizer state (◇) and one probabilistic state (○). Each state is annotated with its priority. Each edge is annotated with a reward by which the energy level is increased after traversing it (respectively, decreased if the reward is negative). The maximizer wins if the lowest priority visited infinitely often is even and the energy level never drops below 0.

view this process as a random walk on a half line. If $x_n$ is the probability of reaching energy level 2 when starting at energy $n$ then these probabilities are the least point-wise positive solution to the following system of linear equations: $x_2 = 1$, $x_n = \frac{2}{3}x_{n+1} + \frac{1}{3}x_{n-1}$ for all $n \geq 3$. We then get that $x_n = \frac{1}{2^{n-2}}$ so the probability of not reaching energy level 2 is $\geq \frac{1}{2}$ for all $n \geq 3$. Always turning left guarantees that, almost surely, the parity condition holds and the limes inferior of the energy level is not $-\infty$. We call this condition *Gain*. Strategies for *Gain* can be used when the energy level is sufficiently high (at least 3 in our example) to win with a positive probability.

However, if maximizer plays for Gain and always moves left, then for every initial energy level the chance of eventually dropping the energy down to level 2 is positive, due to the negative cycle. When that happens, the only other option for the maximizer is to move right. There minimizer can 'choose how to lose', via a disjunction of two conditions that we later formalize as *Bailout*. Either minimizer goes back to the start state without changing the energy level (thus maximizer wins as the energy stays at level 2 and only the good priority 2 is seen), or minimizer turns right. In the latter case, the play visits a dominating odd priority (which is bad for maximizer) but also increases the energy by 1, which allows maximizer to switch back to playing left for the *Gain* condition until energy level 2 is reached again.

Our maximizer strategies are a complex interplay between *Bailout* and *Gain*. In the example, it is easy to see that the probability of seeing priority 1 infinitely often is zero if maximizer follows the just described strategy (the probability of requiring to go right more than $n$ times is at most $(\frac{1}{2})^n$), so maximizer wins this energy-parity game almost surely. Note that maximizer does not win almost surely when the initial energy level is 0 or 1.

**Previous work on combined objectives.** Non-stochastic energy-parity games have been studied in [16]. They can be solved in $\mathsf{NP} \cap \mathsf{coNP}$ and maximizer strategies require only finite (but exponential) memory, a property that also allowed to show P-time inter-reducibility with mean-payoff parity games. More recently they were also shown to be solvable in pseudo-quasi-polynomial time [26]. Related results on non-stochastic games (e.g., mean-payoff parity) are summarized in [18].

Most existing work on combined objectives for stochastic systems, for example [17,18,9,40], is restricted to Markov decision processes (MDPs; aka $1\frac{1}{2}$-player games). Almost-sure energy-parity objectives for MDPs were first considered in [17,18], where a direct reduction to ordinary energy games was proposed. This reduction relies on the assumption that maximizer can win using finite memory if at all. Unfortunately, this assumption does not necessarily hold: it was shown in [40] that an almost sure winning strategy for energy-parity in finite MDPs may require infinite memory. Nevertheless, it was possible to recover the original result, that deciding the existence of a.s. winning strategies is in $\mathsf{NP} \cap \mathsf{coNP}$ (and pseudo-polynomial time), by showing that the existence of an a.s. winning strategy can be witnessed by the existence of two compatible, and finite-memory, winning strategies for two simpler objectives. We generalize this approach from MDPs to full stochastic games.

Stochastic mean-payoff parity games were studied in [20], where it was shown that they can be solved in $\mathsf{NP} \cap \mathsf{coNP}$. However, this does not imply a solution for stochastic energy-parity games, since, unlike in the non-stochastic case [16], there is no known reduction from energy-parity to mean-payoff parity in stochastic games. (The reduction in [16] relies on the fact that maximizer has a winning finite-memory strategy for energy-parity, which does not generally hold for stochastic games or MDPs; see above.)

A related model are the 1-counter MDPs (and stochastic games) studied in [12,11,8], since the value of the counter can be interpreted as the stored energy. These papers consider the objective of reaching counter value zero (which is dual to the energy objective of staying above zero), thus the roles of minimizer and maximizer are swapped. However, unlike in this paper, these works do not combine termination objectives with extra parity conditions.

**Structure of the paper.** The rest of the paper is organized as follows. We start by introducing the notation and formal definitions of games and objectives in the next section. In Section 3 we show how checking almost-sure energy-parity objectives can be characterized in terms of two newly defined auxiliary objectives: Gain and Bailout. In Sections 4 and 5, we show that almost-sure Bailout and Gain objectives, respectively, can be checked in $\mathsf{NP}$ and $\mathsf{coNP}$. Section 6 contains our main result: $\mathsf{NP}$ and $\mathsf{coNP}$ algorithms for checking almost-sure energy-parity games with a known and unknown initial energy, as well as checking if a given initial energy is the minimal one. We conclude and point out some open problems in Section 7. Due to page restrictions, most proofs in the main body of the paper were replaced by sketches. The detailed proofs can be found in the full version of this paper [41].

### 2 Preliminaries

A probability distribution over a set $X$ is a function $f : X \to [0,1]$ such that $\sum_{x \in X} f(x) = 1$. We write $\mathcal{D}(X)$ for the set of distributions over $X$.

**Games, Strategies, Measures.** A *Simple Stochastic Game (SSG)* is a directed graph $\mathcal{G} \stackrel{\text{def}}{=} (V, E, \lambda)$, where all states have an outgoing edge and the set of states is partitioned into states owned by *maximizer* ($V_\Box$), *minimizer* ($V_\Diamond$) and probabilistic states ($V_\bigcirc$). The set of *edges* is $E \subseteq V \times V$ and $\lambda : V_\bigcirc \to \mathcal{D}(E)$ assigns each probabilistic state a probability distribution over its outgoing edges. W.l.o.g., we assume that each probabilistic state has at most two successors, because one can introduce a new probabilistic state for each excess successor. We let $\lambda(ws) \stackrel{\text{def}}{=} \lambda(s)$ for all $ws \in (VE)^* V_\bigcirc$.

A *path* is a finite or infinite sequence $\rho \stackrel{\text{def}}{=} s_0 e_0 s_1 e_1 \ldots$ such that $e_i = (s_i, s_{i+1}) \in E$ holds for all indices $i$. A *run* is an infinite path and we write $\mathit{Runs} \stackrel{\text{def}}{=} (VE)^\omega$ for the set of all runs.

A *strategy* for maximizer is a function $\sigma : (VE)^* V_\Box \to \mathcal{D}(E)$ that assigns to each path $ws \in (VE)^* V_\Box$ a probability distribution over the outgoing edges of its target node $s$. That is, $\sigma(ws)(e) > 0$ implies $e = (s,t) \in E$ for some $t \in V$. A strategy is called *memoryless* if $\sigma(xs) = \sigma(ys)$ for all $x, y \in (VE)^*$ and $s \in V_\Box$, *deterministic* if $\sigma(w)$ is Dirac for all $w \in (VE)^* V_\Box$, and *finite-state* if there exists an equivalence relation $\sim$ on $(VE)^* V_\Box$ with a finite index, such that $\sigma(\rho_1) = \sigma(\rho_2)$ if $\rho_1 \sim \rho_2$. Of particular interest to us will be the class of *memoryless deterministic strategies* (*MD*) and the class of *finite-memory deterministic strategies* (*FD*). Strategies for minimizer are defined analogously and will usually be denoted by $\tau : (VE)^* V_\Diamond \to \mathcal{D}(E)$.

A maximizing (minimizing) *Markov Decision Process (MDP)* is a game in which minimizer (maximizer) has no choices, i.e., all her states have exactly one successor. We will write $\mathcal{G}[\tau]$ for the MDP resulting from fixing the strategy $\tau$. A *Markov chain* is a game where neither player has a choice. In particular, $\mathcal{G}[\sigma, \tau]$ is a Markov chain obtained by setting, in the game $\mathcal{G}$, the strategies for maximizer and minimizer to $\sigma$ and $\tau$, respectively.

Given an initial state $s \in V$ and strategies $\sigma$ and $\tau$ for maximizer and minimizer, respectively, the set of runs starting in $s$ naturally extends to a probability space as follows. We write $Runs_w^{\mathcal{G}}$ for the *w-cylinder*, i.e., the set of all runs with prefix $w \in (VE)^* V$. We let $\mathcal{F}^{\mathcal{G}}$ be the $\sigma$-algebra generated by all these cylinders. We inductively define a probability function $\mathbb{P}_s^{\mathcal{G},\sigma,\tau}$ on all cylinders, which then uniquely extends to $\mathcal{F}^{\mathcal{G}}$ by Carathéodory's extension theorem [5], by setting $\mathbb{P}_s^{\mathcal{G},\sigma,\tau}(Runs_s^{\mathcal{G}}) \overset{\text{def}}{=} 1$ and $\mathbb{P}_s^{\mathcal{G},\sigma,\tau}(Runs_w^{\mathcal{G}}) \overset{\text{def}}{=} \prod_{i=0}^{n-1} dist_i(s_0 e_0 s_1 e_1 \ldots s_i)(e_i)$ for $w = s_0 e_0 s_1 e_1 \ldots e_{n-1} s_n$, where $s_0 = s$, $e_i = (s_i, s_{i+1})$ and $dist_i$ is $\sigma(\cdot)$, $\tau(\cdot)$ or $\lambda(\cdot)$, for $s_i \in V_\Box, V_\Diamond$ or $V_\bigcirc$, respectively.

**Objective Functions.** A (Borel) *objective* is a set $\mathsf{Obj} \in \mathcal{F}^{\mathcal{G}}$ of runs. We write $\overline{\mathsf{Obj}} \overset{\text{def}}{=} Runs \setminus \mathsf{Obj}$ for its complement. Borel objectives $\mathsf{Obj}$ are weakly determined [39,38], which means that

$$\sup_\sigma \inf_\tau \mathbb{P}_s^{\sigma,\tau}(\mathsf{Obj}) = \inf_\tau \sup_\sigma \mathbb{P}_s^{\sigma,\tau}(\mathsf{Obj}).$$

This quantity is called the *value* of $\mathsf{Obj}$ in state $s$, and written as $\mathsf{Val}_s^{\mathcal{G}}(\mathsf{Obj})$. We say that $\mathsf{Obj}$ holds *almost-surely* (abbreviated as *a.s.*) at state $s$ iff there exists $\sigma$ such that $\forall \tau, \mathbb{P}_s^{\mathcal{G},\sigma,\tau}(\mathsf{Obj}) = 1$. Let $\mathsf{AS}^{\mathcal{G}}(\mathsf{Obj})$ denote the set of states at which $\mathsf{Obj}$ holds almost surely. We will drop the superscript $\mathcal{G}$ and simply write *Runs*, $\mathbb{P}_s^{\sigma,\tau}$ and $\mathsf{AS}(\mathsf{Obj})$, if the game is clear from the context.

We use the syntax and semantics of operators $\mathbb{F}$ (eventually) and $\mathbb{G}$ (always) from the temporal logic LTL [25] to specify some conditions on runs.

A *reachability condition* is defined by a set of target states $T \subseteq V$. A run $\rho = s_0 e_0 s_1 \ldots$ satisfies the reachability condition iff there exists an $i \in \mathbb{N}$ s.t. $s_i \in T$. We write $\mathbb{F}T \subseteq Runs$ for the set of runs that satisfy this reachability condition. Given a set of states $W \subseteq V$, we lift this to a safety condition on runs and write $\mathbb{G}W \subseteq Runs$ for the set of runs $\rho = s_0 e_0 s_1 \ldots$ where $\forall i.\, s_i \in W$.

A *parity condition* is given by a bounded function $parity : V \to \mathbb{N}$ that assigns a priority (a non-negative integer) to each state. A run $\rho \in Runs$ satisfies the parity condition iff the minimal priority that appears infinitely often on the run is even. The *parity objective* is the subset $\mathsf{PAR} \subseteq Runs$ of runs that satisfy the parity condition.

*Energy conditions* are given by a function $r : E \to \mathbb{Z}$, that assigns a *reward* value to each edge. For a given initial energy value $k \in \mathbb{N}$, a run $s_0 e_0 s_1 e_1 \ldots$ satisfies the $k$-energy condition if, for every finite prefix of length $n$, the *energy level* $k + \sum_{i=0}^{n} r(e_i)$ is greater or equal to 0. Let $\mathsf{EN}(k) \subseteq Runs$ denote the $k$-energy objective, consisting of those runs that satisfy the $k$-energy condition.

The *$l$-storage condition* holds for a run $s_0 e_0 s_1 e_1 \ldots$ if $l + \sum_{i=m}^{n-1} r(s_i, s_{i+1}) \geq 0$ holds for every infix $s_m e_m s_{m+1} \ldots s_n$. Let $\mathsf{ST}(k,l) \subseteq Runs$ denote the $k$-energy $l$-storage objective, consisting of those runs that satisfy both the $k$-energy and the $l$-storage condition. We write $\mathsf{ST}(k)$ for $\bigcup_l \mathsf{ST}(k,l)$. Clearly, $\mathsf{ST}(k) \subseteq \mathsf{EN}(k)$.

*Mean-payoff* and *limit-payoff conditions* are defined w.r.t. the same reward function as the energy conditions. The *mean-payoff* value of a run $\rho = s_0 e_0 s_1 e_1 \ldots$ is $MP(\rho) \stackrel{\text{def}}{=} \liminf_{n\to\infty} \frac{1}{n} \sum_{i=0}^{n-1} r(e_i)$. For $\triangle \in \{>, \geq, =, \leq, <\}$ and $c \in \mathbb{R} \cup \{-\infty, \infty\}$, the set $\mathsf{MP}(\triangle c) \subseteq Runs$ consists of all runs $\rho$ with $MP(\rho) \triangle c$. Let $\mathsf{LimInf}(\triangle c) \subseteq Runs$ contain all runs $\rho$ with $(\liminf_{n\to\infty} \sum_{i=0}^{n} r(e_i)) \triangle c$, and likewise for $\mathsf{LimSup}(\triangle c)$.

The combined energy-parity objective $\mathsf{EN}(k) \cap \mathsf{PAR}$ is Borel and therefore weakly determined, meaning that it has a well-defined (inf sup = sup inf) value for every game [39,38]. Moreover, the almost-sure energy-parity objective (asking to win with probability 1) is even strongly determined [37]: either maximizer has a strategy to enforce the condition with probability 1 or minimizer has a strategy to prevent this.

### 3 Characterizing Energy-Parity via Gain and Bailout

The main theorem of this section (Theorem 5) characterizes almost sure energy-parity objectives in terms of two intermediate objectives called $\mathsf{Gain}$ and $k$-$\mathsf{Bailout}$ for parameters $k \geq 0$. This will form the basis of all computability results: we will show (as Theorems 14, 17 and 18) how to compute almost-sure sets for these intermediate objectives.

**Definition 2.** *Consider a finite SSG $\mathcal{G} = (V, E, \lambda)$, as well as reward and parity functions defining the objectives* $\mathsf{PAR}, \mathsf{LimInf}(> -\infty), \mathsf{LimSup}(= \infty)$ *as well as* $\mathsf{ST}(k,l)$ *and* $\mathsf{EN}(k)$ *for every $k, l \in \mathbb{N}$. We define combined objectives* $\mathsf{Gain}$ *and* $k$-$\mathsf{Bailout} \stackrel{def}{=} \cup_l \mathsf{Bailout}(k,l)$ *where*

$$\begin{aligned} \mathsf{Gain} &\stackrel{def}{=} \mathsf{LimInf}(> -\infty) \cap \mathsf{PAR} \\ \mathsf{Bailout}(k,l) &\stackrel{def}{=} (\mathsf{ST}(k,l) \cap \mathsf{PAR}) \cup (\mathsf{EN}(k) \cap \mathsf{LimSup}(= \infty)). \end{aligned}$$

The main idea behind these two objectives is a special witness property for energy-parity. We argue that, if maximizer has an almost-sure winning strategy for energy-parity then he also has one that combines two almost-sure winning strategies, one for $\mathsf{Gain}$ and one for $k$-$\mathsf{Bailout}$.

Notice that playing an almost-sure winning strategy for $\mathsf{Gain}$ implies a uniformly lower-bounded strictly positive chance that the energy level never drops below zero (assuming it is sufficiently high to begin with). This fact uses the finiteness of the set of control-states and does not hold for infinite-state MDPs. In the unlikely event that the energy level does get close to zero, maximizer switches to playing an almost sure winning strategy for $k$-$\mathsf{Bailout}$. This is a disjunction of two scenarios, and the balance might be influenced by minimizer's choices. In the first scenario ($\mathsf{ST}(k,l) \cap \mathsf{PAR}$) the energy never drops much and stays above zero (thus satisfying energy-parity). In the second scenario, ($\mathsf{EN}(k) \cap \mathsf{LimSup}(= \infty)$), the parity objective is temporarily suspended in favor of boosting (while always staying above zero) the energy to a sufficiently high level to switch back to the strategy for $\mathsf{Gain}$ and thus try again from the beginning. The probability of infinitely often switching between these modes is zero due to the lower-bounded chance of success in the $\mathsf{Gain}$ phase. Therefore, maximizer eventually wins by playing for $\mathsf{Gain}$. Note that maximizer needs to remember the current energy level in order to know when to switch and consequently, this strategy uses infinite memory.

*Example 3.* Consider again the game in Fig. 1. The middle left state satisfies both $\mathsf{Gain}$ and $k$-$\mathsf{Bailout}$ objectives for all $k \geq 2$ almost-surely. The respective winning strategies are to always go left for $\mathsf{Gain}$ or always go right for $k$-$\mathsf{Bailout}$ when at that state. Note that it neither satisfies 0-$\mathsf{Bailout}$ nor 1-$\mathsf{Bailout}$ objectives.

We define the subset $W \subseteq V$ of states from which maximizer can almost surely win both $\mathsf{Gain}$ and $k$-$\mathsf{Bailout}$ (assuming sufficiently high initial energy), while at the same time ensuring that the play remains within this set of states. These are the states from which maximizer can win by freely combining individual strategies for the $\mathsf{Gain}$ and $\mathsf{Bailout}$ objectives.

**Definition 4.** *Given a finite SSG $\mathcal{G} = (V, E, \lambda)$, let $W \subseteq V$ be the largest subset of states satisfying the following condition*

$$W \subseteq \mathsf{AS}\,(\mathsf{Gain} \cap \mathbb{G}W) \cap \bigcup_k \mathsf{AS}\,(k\text{-}\mathsf{Bailout} \cap \mathbb{G}W)$$

This condition describes a fixed-point, and as it is easy to see that if two sets $W_1$ and $W_2$ are such fixed-points, then so is $W_1 \cup W_2$. Thus, the maximal fixed-point $W$ is well-defined.

Our main characterization of almost-sure energy-parity objectives is the following Theorem 5. It states that maximizer can almost surely win an $\mathsf{EN}(k) \cap \mathsf{PAR}$ objective if, and only if, he can win the easier $k$-Bailout objective while always staying in the safe set $W$.

**Theorem 5.** *For every $k \in \mathbb{N}$, $\mathsf{AS}\,(\mathsf{EN}(k) \cap \mathsf{PAR}) = \mathsf{AS}\,(k\text{-}\mathsf{Bailout} \cap \mathbb{G}W)$.*

Our proof of this characterization theorem relies on the following claim, which allows to lift the existence of finite-memory deterministic optimal strategies from MDPs to SSGs. It applies to a fairly general class of objectives and, we believe, is of independent interest.

Recall that $\overline{\mathsf{Obj}} \stackrel{\text{def}}{=} \mathit{Runs} \setminus \mathsf{Obj}$ denotes the complement of objective $\mathsf{Obj}$. For runs $a, b, c \in \mathit{Runs}$ we say that $a$ is a *shuffle* of $b$ and $c$ if there exist factorizations $b = b_0 b_1 \ldots$ and $c = c_0 c_1 \ldots$ such that $a = b_0 c_0 b_1 c_1 \ldots$. An objective $\mathsf{Obj}$ is called *submixing* if, for every run $a \in \mathsf{Obj}$ that is a shuffle of runs $b$ and $c$, either $b \in \mathsf{Obj}$ or $c \in \mathsf{Obj}$. $\mathsf{Obj}$ is *shift-invariant* if, for every run $s_1 e_1 s_2 e_2 \ldots$, it holds that $s_1 e_1 s_2 e_2 \ldots \in \mathsf{Obj} \iff s_2 e_2 \ldots \in \mathsf{Obj}$. Shift-invariance slightly generalizes the better-known *tail* condition (see [34] for a discussion).

**Theorem 6.** *Let $\mathcal{O}$ be an objective such that $\overline{\mathcal{O}}$ is both shift-invariant and submixing. If maximizer has optimal FD strategies (from any state s) for $\mathcal{O}$ for every finite MDP then maximizer has optimal FD strategies (from any state s) for $\mathcal{O}$ for every finite SSG.*

This applies in particular to the $\mathsf{Gain}$ objective, but not to $k$-$\mathsf{Bailout}$ objectives, as these are not shift-invariant. A proof of Theorem 6 can be found in [41]. It uses a recursive argument based on the notion of *reset strategies* from [34].

The remainder of this section is dedicated to proving Theorem 5. We will first collect the remaining technical claims about $\mathsf{Gain}$, $\mathsf{Bailout}$, and reachability objectives. Most notably, as Lemma 8, we show that if maximizer can almost surely win $\mathsf{Gain}$ in a SSG, then he can do so using a FD strategy which moreover satisfies an energy-parity objective with strictly positive (and lower-bounded) probability. This is shown in part based on Theorem 6 applied to the $\mathsf{Gain}$ objective. We will also need the following fact about reachability objectives in finite MDPs.

**Lemma 7 ([8, Lemma 3.9]).** *Let $\mathcal{M}$ be a finite MDP and $\mathit{Reach}_T$ be the reachability objective with target $T \stackrel{\text{def}}{=} \{s' \mid \mathsf{Val}_{s'}(\mathsf{LimInf}(= -\infty)) = 1\}$. One can compute a rational constant $c < 1$ and an integer $h \geq 0$ such that for all states $s$ and $i \geq h$ we have $\forall \tau. \mathbb{P}_s^{\tau}(\overline{\mathsf{EN}(i)} \cap \overline{\mathit{Reach}_T}) \leq \frac{c^i}{1-c}$.*

**Lemma 8.** *Consider a finite SSG $\mathcal{G} = (V, E, \lambda)$ where $\mathsf{Gain}$ holds a.s. for every state $s \in V$. Then, for every $\delta \in [0, 1)$ and $s \in V$, there exists a $\hat{k} \in \mathbb{N}$ and an FD strategy $\hat{\sigma}$ s.t.*

1. $\forall \tau. \mathbb{P}_s^{\hat{\sigma},\tau}(\mathsf{Gain}) = 1$, *and*
2. $\forall \tau. \mathbb{P}_s^{\hat{\sigma},\tau}(\mathsf{EN}(\hat{k}) \cap \mathsf{PAR}) \geq \delta$.

*Proof.* Fix a $\delta \in [0, 1)$ and a state $s \in V$. Both $\mathsf{LimInf}(= -\infty)$, as well as $\mathsf{PAR}$ objectives are *shift-invariant* and *submixing*, and therefore also the union has both these properties. It follows that $\overline{\mathsf{Gain}} = \overline{\mathsf{LimInf}(> -\infty) \cap \mathsf{PAR}} = \mathsf{LimInf}(= -\infty) \cup \overline{\mathsf{PAR}}$ is both shift-invariant and submixing, since the complement of a parity objective is also a parity objective. By Lemma 16 and Theorem 6, there exists an almost-sure winning FD strategy $\hat{\sigma}$ for maximizer for the objective $\mathsf{Gain}$ from $s$, i.e., $\forall \tau.\, \mathbb{P}_s^{\hat{\sigma},\tau}(\mathsf{Gain}) = 1$, thus yielding Item 1.

Let $\mathcal{M}$ be the MDP obtained from $\mathcal{G}$ by fixing the strategy $\hat{\sigma}$ for maximizer from $s$. Since $\mathcal{G}$ is finite and $\hat{\sigma}$ is FD, also $\mathcal{M}$ is finite. In $\mathcal{M}$ we have $\forall \tau.\, \mathbb{P}_s^{\tau}(\mathsf{Gain}) = 1$. In particular, in $\mathcal{M}$, the set $T \stackrel{\text{def}}{=} \{s' \mid \mathsf{Val}_{s'}(\mathsf{LimInf}(= -\infty)) = 1\}$ is not reachable, i.e., $\forall \tau.\, \mathbb{P}_s^{\tau}(\mathit{Reach}_T) = 0$.

By Lemma 7, in $\mathcal{M}$ there exists a horizon $h \in \mathbb{N}$ and a constant $c < 1$ such that for all $i \geq h$ we have $\forall \tau.\, \mathbb{P}_s^{\tau}(\overline{\mathsf{EN}(i)} \cap \overline{\mathit{Reach}_T}) \leq \frac{c^i}{1-c}$. Since $T$ cannot be reached in $\mathcal{M}$, the condition $\overline{\mathit{Reach}_T}$ evaluates to *true* and we have $\forall \tau.\, \mathbb{P}_s^{\tau}(\mathsf{EN}(i)) \geq 1 - \frac{c^i}{1-c}$. Since $c < 1$ and $\delta < 1$, we can pick a sufficiently large $\hat{k} \geq h$ such that $1 - \frac{c^{\hat{k}}}{1-c} \geq \delta$ and obtain $\forall \tau.\, \mathbb{P}_s^{\tau}(\mathsf{EN}(\hat{k})) \geq \delta$ in $\mathcal{M}$. Moreover, the above property $\forall \tau.\, \mathbb{P}_s^{\tau}(\mathsf{Gain}) = 1$ in particular implies $\forall \tau.\, \mathbb{P}_s^{\tau}(\mathsf{PAR}) = 1$. Thus we obtain $\forall \tau.\, \mathbb{P}_s^{\tau}(\mathsf{EN}(\hat{k}) \cap \mathsf{PAR}) \geq \delta$ in $\mathcal{M}$.

Back in the SSG $\mathcal{G}$, we have $\forall \tau.\, \mathbb{P}_s^{\hat{\sigma},\tau}(\mathsf{EN}(\hat{k}) \cap \mathsf{PAR}) \geq \delta$ as required for Item 2. □

**Lemma 9.** *$\mathsf{EN}(k) \cap \mathsf{PAR} \subseteq k$-$\mathsf{Bailout}$.*

*Proof.* Let $\rho$ be a run in $\mathsf{EN}(k) \cap \mathsf{PAR}$. There are two cases. In the first case we have $\rho \in \cup_l \mathsf{ST}(k,l) \cap \mathsf{PAR}$ and thus directly $\rho \in k$-$\mathsf{Bailout}$. Otherwise, $\rho \notin \cup_l \mathsf{ST}(k,l) \cap \mathsf{PAR}$. Since $\rho \in \mathsf{PAR}$, we must have $\rho \notin \cup_l \mathsf{ST}(k,l)$. Since $\rho \in \mathsf{EN}(k)$, it follows that $\rho$ does not satisfy the $l$-storage condition for any $l \in \mathbb{N}$. So, for every $l \in \mathbb{N}$, there exists an infix $\rho'$ of $\rho$ s.t. $l + r(\rho') < 0$. Let $\rho''$ be the prefix of $\rho$ before $\rho'$. Since $\rho \in \mathsf{EN}(k)$ we have $k + r(\rho''\rho') \geq 0$ and thus $r(\rho'') \geq -k - r(\rho') > -k + l$. To summarize, if $\rho \notin \cup_l \mathsf{ST}(k,l) \cap \mathsf{PAR}$ then, for every $l$, it has a prefix $\rho''$ with $r(\rho'') > -k + l$. Thus $\rho \in \mathsf{LimSup}(= \infty)$. Thus $\rho \in k$-$\mathsf{Bailout}$. □

We now define $W'$ as the set of states that are almost-sure winning for energy-parity with some sufficiently high initial energy level. ($W'$ is also called the winning set for the unknown initial credit problem.)

**Definition 10.** $W' \stackrel{\text{def}}{=} \bigcup_k \mathsf{AS}\,(\mathsf{EN}(k) \cap \mathsf{PAR})$.

**Lemma 11.**

1. $\mathsf{AS}\,(\mathsf{EN}(k) \cap \mathsf{PAR}) \subseteq \mathsf{AS}\,(\mathsf{Gain} \cap \mathbb{G} W')$
2. $\mathsf{AS}\,(\mathsf{EN}(k) \cap \mathsf{PAR}) \subseteq \mathsf{AS}\,(k\text{-}\mathsf{Bailout} \cap \mathbb{G} W')$

*Proof.* Let $s \in \mathsf{AS}\,(\mathsf{EN}(k) \cap \mathsf{PAR})$ and $\sigma$ a strategy that witnesses this property. Except for a null-set, all runs $\rho = s e_0 s_1 e_1 \dots e_{n-1} s_n \dots$ from $s$ induced by $\sigma$ satisfy $\mathsf{EN}(k) \cap \mathsf{PAR}$.

Let $\rho' = s e_0 s_1 e_1 \dots s_m$ be a finite prefix of $\rho$. For every $n \geq 0$ we have $k + \sum_{i=0}^{n-1} r(e_i) \geq 0$, since $\rho \in \mathsf{EN}(k)$. In particular this holds for all $n \geq m$. So, for every $n \geq m$, we have $k + \sum_{i=0}^{m-1} r(e_i) + \sum_{i=m}^{n-1} r(e_i) \geq 0$. Therefore $s_m \in \mathsf{AS}\,(\mathsf{EN}(k') \cap \mathsf{PAR})$, where $k' = k + \sum_{i=0}^{m-1} r(e_i)$, as witnessed by playing $\sigma$ with history $s e_0 s_1 e_1 \dots s_m$ from $s_m$. Thus $s_m \in \bigcup_k \mathsf{AS}\,(\mathsf{EN}(k) \cap \mathsf{PAR}) = W'$, i.e., almost all $\sigma$-induced runs $\rho$ satisfy $\mathbb{G} W'$.

Towards Item 1, we have $\mathsf{EN}(k) \subseteq \mathsf{LimInf}(> -\infty)$ and thus $\mathsf{EN}(k) \cap \mathsf{PAR} \subseteq \mathsf{LimInf}(> -\infty) \cap \mathsf{PAR} = \mathsf{Gain}$. Therefore $\sigma$ witnesses $s \in \mathsf{AS}\,(\mathsf{Gain} \cap \mathbb{G}W')$.

Towards Item 2, we have $\mathsf{EN}(k) \cap \mathsf{PAR} \subseteq k\text{-}\mathsf{Bailout}$ by Lemma 9. Thus $\sigma$ witnesses $s \in \mathsf{AS}\,(k\text{-}\mathsf{Bailout} \cap \mathbb{G}W')$. □

**Lemma 12.** *$W' \subseteq W$.*

*Proof.* It suffices to show that $W'$ satisfies the monotone condition imposed on $W$ (cf. Definition 4), since $W$ is defined as the largest set satisfying this condition.

Let $s \in W' = \bigcup_k \mathsf{AS}\,(\mathsf{EN}(k) \cap \mathsf{PAR})$. Then $s \in \mathsf{AS}\left(\mathsf{EN}(\hat{k}) \cap \mathsf{PAR}\right)$ for some fixed $\hat{k}$. By Lemma 11(1) we have $s \in \mathsf{AS}\,(\mathsf{Gain} \cap \mathbb{G}W')$. By Lemma 11(2) we have $s \in \mathsf{AS}\left(\hat{k}\text{-}\mathsf{Bailout} \cap \mathbb{G}W'\right) \subseteq \bigcup_k \mathsf{AS}\,(k\text{-}\mathsf{Bailout} \cap \mathbb{G}W')$. □

*Proof of Theorem 5.* Towards the $\subseteq$ inclusion, we have

$$\mathsf{AS}\,(\mathsf{EN}(k) \cap \mathsf{PAR}) \subseteq \mathsf{AS}\,(k\text{-}\mathsf{Bailout} \cap \mathbb{G}W') \subseteq \mathsf{AS}\,(k\text{-}\mathsf{Bailout} \cap \mathbb{G}W)$$

by Lemma 11(2) and Lemma 12.

Towards the $\supseteq$ inclusion, let $s \in \mathsf{AS}\,(k\text{-}\mathsf{Bailout} \cap \mathbb{G}W)$ and $\sigma_1$ be a strategy that witnesses this. We show that $s \in \mathsf{AS}\,(\mathsf{EN}(k) \cap \mathsf{PAR})$. We now consider the modified SSG $\mathcal{G}' = (W, E, \lambda)$ with the state set restricted to $W$. In particular, $s \in W$ and $\sigma_1$ witnesses $s \in \mathsf{AS}\,(k\text{-}\mathsf{Bailout})$ in $\mathcal{G}'$. We now construct a strategy $\sigma$ that witnesses $s \in \mathsf{AS}\,(\mathsf{EN}(k) \cap \mathsf{PAR})$ in $\mathcal{G}'$, and thus also in $\mathcal{G}$. The strategy $\sigma$ will use infinite memory to keep track of the current energy level of the run.

Apart from $\sigma_1$, we require several more strategies as building blocks for the construction of $\sigma$.

First, in $\mathcal{G}$ we had $\forall s' \in W.\, s' \in \mathsf{AS}\,(\mathsf{Gain} \cap \mathbb{G}W)$, and thus in $\mathcal{G}'$ we have $\forall s' \in W.\, s' \in \mathsf{AS}\,(\mathsf{Gain})$. For every $s' \in W$ we instantiate Lemma 8 for $\mathcal{G}'$ with $\delta = 1/2$ and obtain a number $\hat{k}_{s'}$ and a strategy $\hat{\sigma}_{s'}$ with

1. $\forall \tau.\, \mathbb{P}_{s'}^{\hat{\sigma}_{s'},\tau}(\mathsf{Gain}) = 1$, and
2. $\forall \tau.\, \mathbb{P}_{s'}^{\hat{\sigma}_{s'},\tau}(\mathsf{EN}(\hat{k}_{s'}) \cap \mathsf{PAR}) \geq 1/2$.

Let $k_1 \overset{\text{def}}{=} \max\{\hat{k}_{s'} \mid s' \in W\}$. The strategies $\hat{\sigma}_{s'}$ are called *gain strategies*.

Second, by the finiteness of $V$, there is a minimal number $k_2$ such that $\bigcup_k \mathsf{AS}\,(k\text{-}\mathsf{Bailout} \cap \mathbb{G}W) = \bigcup_{k \leq k_2} \mathsf{AS}\,(k\text{-}\mathsf{Bailout} \cap \mathbb{G}W)$ in $\mathcal{G}$. Therefore, in $\mathcal{G}'$ we have that

$$W \subseteq \bigcup_k \mathsf{AS}\,(k\text{-}\mathsf{Bailout}) = \bigcup_{k \leq k_2} \mathsf{AS}\,(k\text{-}\mathsf{Bailout}) = \mathsf{AS}\,(k_2\text{-}\mathsf{Bailout})\,.$$

Thus in $\mathcal{G}'$ for every $s' \in W$ there exists a strategy $\tilde{\sigma}_{s'}$ with $\forall \tau.\, \mathbb{P}_{s'}^{\tilde{\sigma}_{s'},\tau}(k_2\text{-}\mathsf{Bailout}) = 1$. The strategies $\tilde{\sigma}_{s'}$ are called *bailout strategies*. Let $k' \overset{\text{def}}{=} k_1 + k_2 - k + 1$. We now define the strategy $\sigma$.

**Start:** First $\sigma$ plays like $\sigma_1$ from $s$. Since $\sigma_1$ witnesses $s \in \mathsf{AS}\,(k\text{-}\mathsf{Bailout})$ against every minimizer strategy $\tau$, almost all induced runs $\rho = s e_0 s_1 e_1 \ldots$ satisfy either

**(A)** $(\cup_l \mathsf{ST}(k, l) \cap \mathsf{PAR})$, or

**(B)** $(\mathsf{EN}(k) \cap \mathsf{LimSup}(= \infty))$.

Almost all runs $\rho$ of the latter type (B) (and potentially also some runs of type (A)) satisfy $\mathsf{EN}(k)$ and $\sum_{i=0}^{l} r(e_i) \geq k'$ eventually for some $l$. If we observe $\sum_{i=0}^{l} r(e_i) \geq k'$ for some prefix $se_0s_1e_1 \ldots e_ls'$ of the run $\rho$ then our strategy $\sigma$ plays from $s'$ as described in the **Gain** part below. Otherwise, if we never observe this condition, then our run $\rho$ is of type (A) and $\sigma$ continues playing like $\sigma_1$. Since property (A) implies $(\mathsf{EN}(k) \cap \mathsf{PAR})$, this is sufficient.

**Gain:** In this case we are in the situation where we have reached some state $s'$ after some finite prefix $\rho'$ of the run, where $r(\rho') \geq k'$. Our strategy $\sigma$ now plays like the gain strategy $\hat{\sigma}_{s'}$, as long as $r(\rho') \geq k' - k_1$ holds for the current prefix $\rho'$ of the run. By Item 2, this will satisfy $\forall \tau. \mathbb{P}_{s'}^{\hat{\sigma}_{s'}, \tau}(\mathsf{EN}(\hat{k}_{s'}) \cap \mathsf{PAR}) \geq 1/2$ and thus $\forall \tau. \mathbb{P}_{s'}^{\hat{\sigma}_{s'}, \tau}(\mathsf{EN}(k_1) \cap \mathsf{PAR}) \geq 1/2$. It follows that with probability $\geq 1/2$ we will keep playing $\hat{\sigma}_{s'}$ forever and satisfy $\mathsf{PAR}$ and always $r(\rho') \geq k' - k_1$ and thus $\mathsf{EN}(k)$, since $k + r(\rho') \geq k + k' - k_1 = k_2 + 1 \geq 0$.

Otherwise, if eventually $r(\rho') = k' - k_1 - 1$ then we have $k + r(\rho') = k_2$. In this case (which happens with probability $< 1/2$) we continue playing as described in the **Bailout** part below.

**Bailout:** In this case we are in the situation where we have reached some state $s'' \in W$ after some finite prefix $\rho'$ of the run, where $k + r(\rho') = k_2$. Since $s'' \in W$, we can now let our strategy $\sigma$ play like the bailout strategy $\tilde{\sigma}_{s''}$ and obtain $\forall \tau. \mathbb{P}_{s''}^{\tilde{\sigma}_{s''}, \tau}(k_2\text{-}\mathsf{Bailout}) = 1$. Thus almost all induced runs $\rho'' = s''e_0s_1e_1 \ldots$ from $s''$ satisfy either

**(A)** $(\cup_l \mathsf{ST}(k_2, l) \cap \mathsf{PAR})$, or

**(B)** $(\mathsf{EN}(k_2) \cap \mathsf{LimSup}(= \infty))$.

As long as $r(\rho') < k'$ holds for the current prefix $\rho'$ of the run, we keep playing $\tilde{\sigma}_{s''}$. Otherwise, if eventually $r(\rho') \geq k'$ holds, then we switch back to playing the **Gain** strategy above. All the runs that never switch back to playing the **Gain** strategy must be of type (A) and thus satisfy $\mathsf{PAR}$. Since we have $k_2\text{-}\mathsf{Bailout} \subseteq \mathsf{EN}(k_2)$, it follows that, for every prefix $\rho''$ of the run from $s''$, according to $\tilde{\sigma}_{s''}$ we have $k_2 + r(\rho'') \geq 0$. Thus, for every prefix $\rho'''$ of $\rho$, we have $k + r(\rho''') = k + r(\rho') + r(\rho'') = k_2 + r(\rho'') \geq 0$. Therefore, the $\mathsf{EN}(k)$ objective is satisfied by all runs.

As shown above, almost all runs induced by $\sigma$ that eventually stop switching between the three modes satisfy $\mathsf{EN}(k) \cap \mathsf{PAR}$. Switching from Gain/Bailout to Start is impossible, but switching from Gain to Bailout and back is possible. However, the set of runs that infinitely often switch between Gain and Bailout is a null-set, because the probability of switching from Gain to Bailout is $\leq 1/2$. Thus, $\sigma$ witnesses $s \in \mathsf{AS}\,(\mathsf{EN}(k) \cap \mathsf{PAR})$. □

*Remark 13.* It follows from the results above that $W' = W$. The $\subseteq$ inclusion holds by Lemma 12. For the reverse inclusion we have

$$\begin{aligned} W &\subseteq \bigcup_k \mathsf{AS}\,(k\text{-}\mathsf{Bailout} \cap \mathbb{G}W) && \text{by Definition 4} \\ &= \bigcup_k \mathsf{AS}\,(\mathsf{EN}(k) \cap \mathsf{PAR}) && \text{by Theorem 5} \\ &= W' && \text{by Definition 10.} \end{aligned}$$

### 4 Bailout

In this section we will argue that it is possible decide, in NP and coNP, whether the bailout objective can be satisfied almost surely. More precisely, we show the existence of procedures to decide if, for a given $k \in \mathbb{N}$ and state $s$, there exists an $l \in \mathbb{N}$ such that $s$ almost-surely satisfies the $\mathsf{Bailout}(k,l)$ objective

$$\mathsf{Bailout}(k,l) \quad \stackrel{\text{def}}{=} \quad (\mathsf{ST}(k,l) \cap \mathsf{PAR}) \cup (\mathsf{EN}(k) \cap \mathsf{LimSup}(=\infty)).$$

Recall that the idea behind the Bailout objective is that, during a game for energy-parity, maximizer is temporarily abandoning the parity (but not the energy) condition in order to increase the energy to a sufficient level (which will then allow him to try an a.s. strategy for Gain once more). However, in a stochastic game – as opposed to an MDP [40] – an opponent could possibly prevent this increase in energy level at the expense of satisfying the original energy-parity objective in the first place (cf. Example 1). The Bailout objective is designed to capture the disjunction of both outcomes, as both are favorable for the maximizer. The parameter $k$ is the acceptable total energy drop (i.e., the initial value), and the parameter $l$ is the acceptable energy drop on any infix of a play, which translates to the upper bound on the energy level in the second outcome.

The question can be phrased equivalently as membership of a control state $s$ in the almost-sure set for the $k$-Bailout objective for a given game $\mathcal{G}$ and energy level $k \in \mathbb{N}$.

**Theorem 14.** *One can check in* NP, coNP *and pseudo-polynomial time if, for a given SSG* $\mathcal{G} \stackrel{\text{def}}{=} (V, E, \lambda)$, $k \in \mathbb{N}$ *and control state* $s \in V$, *maximizer can almost-surely satisfy* $k$-Bailout *from* $s$.

*Moreover, there are* $K, L \in \mathbb{N}$, *polynomial in* $|V|$ *and the largest absolute transition reward, so that* $\bigcup_{k\geq 0} \mathsf{AS}^{\mathcal{G}}\,(k\text{-}\mathsf{Bailout}) = \mathsf{AS}^{\mathcal{G}}\,(\mathsf{Bailout}(K,L))$. *And so, checking whether state* $s$ *belongs to* $\bigcup_{k\geq 0} \mathsf{AS}^{\mathcal{G}}\,(k\text{-}\mathsf{Bailout})$ *is in* NP *and* coNP.

*Proof (sketch).* This is shown by a sequence of transformations of the game and ultimately reduced to a finding the winner of a non-stochastic game with an energy-parity objective, which is known to be solvable in NP, coNP and pseudo-polynomial time [19]. One important observation is that it is possible to replace, without changing the outcome, the energy $\mathsf{EN}(k)$ condition in the $\mathsf{Bailout}(k, l)$ objective by the more restrictive energy-storage $\mathsf{ST}(k, l)$ condition. See [41] for further details. □

## 5 Gain

In this section we will argue that it is possible to decide, in $\mathsf{NP}$ and $\mathsf{coNP}$, whether the $\mathsf{Gain}$ objective (i.e., $\mathsf{LimInf}(> -\infty) \cap \mathsf{PAR}$) can be satisfied almost surely.

We start by investigating the strategy complexity of winning strategies for the $\mathsf{Gain}$ objective.

**Lemma 15.** *In every finite SSG, minimizer has optimal MD strategies for objective* $\mathsf{Gain}$*.*

*Proof.* We show that maximizer has MD optimal strategies for $\mathsf{LimInf}(= -\infty) \cup \mathsf{PAR}$. This is equivalent to the claim of the lemma because $\overline{\mathsf{LimInf}(> -\infty) \cap \mathsf{PAR}} = \mathsf{LimInf}(= -\infty) \cup \overline{\mathsf{PAR}}$ and the complement of a parity condition is itself a parity condition (with all priorities incremented by one).

We note that both $\mathsf{LimInf}(= -\infty)$, as well as parity objectives $\mathsf{PAR}$ are shift-invariant and submixing and therefore also that the union $\mathsf{LimInf}(= -\infty) \cup \mathsf{PAR}$ has both these properties. The claim now follows from the fact that SSGs with objectives that are both submixing and shift-invariant admit MD optimal strategies for maximizer [34, Theorem 5.2]. □

Based on the results in [40] one can show a similar claim for maximizer strategies in MDPs.

**Lemma 16.** *For finite MDPs, almost-sure winning maximizer strategies for* $\mathsf{Gain}$ *can be chosen FD.*

Using the existence of MD optimal minimizer strategies (Lemma 15) and a $\mathsf{coNP}$ upper bound for checking almost sure $\mathsf{Gain}$ in MDPs established in [40], we can derive a $\mathsf{coNP}$ procedure. See [41] for full details.

**Theorem 17.** *Checking whether a state $s \in V$ of a SSG satisfies* $\mathsf{Gain}$ *almost-surely is in* $\mathsf{coNP}$*.*

The rest of this section will deal with the $\mathsf{NP}$ upper bound, which is the most challenging part of this paper. The crux of our proof is the observation that if maximizer has a strategy that wins almost surely against all MD minimizer strategies, then he wins almost surely. This is because one of these MD strategies is optimal due to Lemma 15. We show that, in order to witness such an almost-sure winning strategy for maximizer in SSG $\mathcal{G}$, it suffices to provide a polynomially larger SSG $\mathcal{G}_3$, together with an almost-sure winning strategy for the *storage-parity* objective (see Theorem 21 in Section 6) in $\mathcal{G}_3$. This will give us an $\mathsf{NP}$ algorithm, because $\mathcal{G}_3$, along with its winning strategy, can be guessed and verified in polynomial time. Formally we claim that:

**Theorem 18.** *Checking whether a state* $s \in V$ *of* $\mathcal{G}$ *satisfies* $\mathsf{Gain}$ *almost-surely is in* $\mathsf{NP}$.

*Proof.* (sketch) For technical convenience, we will assume w.l.o.g. that every SSG henceforth is in a normal form, where every random state has only one predecessor, which is owned by the maximizer. To show the existence of $\mathcal{G}_3$, we are going to introduce two intermediate games: $\mathcal{G}_1$ and $\mathcal{G}_2$. These games are never constructed by our $\mathsf{NP}$ algorithm, but are just defined to break down the complex construction of $\mathcal{G}_3$ into more manageable steps.

Intuitively, $\mathcal{G}_1$ is just $\mathcal{G}$ where all rewards on edges are multiplied by a large enough factor, $f$, to turn strategies with a mean-payoff $> 0$ into ones with mean-payoff $> 2$. $\mathcal{G}_2$ is an extension of $\mathcal{G}_1$ where the maximizer is given a choice before every visit to a probabilistic node. He can either let the game proceed as before, or sacrifice part of his one-step reward in exchange for a more evenly balanced reward outcome, so the energy can no longer drop arbitrarily low when a probabilistic cycle is reached. As a result, in $\mathcal{G}_2$ it suffices to consider a storage-parity objective (see Theorem 21 in Section 6) instead of $\mathsf{Gain}$. The number of choices maximizer is given is the number of MD minimizer strategies, which clearly can be exponential. That would not suffice for an $\mathsf{NP}$ algorithm. Therefore, we show that most of these choices are redundant and can be removed without impairing the almost sure wining region. As the result of that pruning, we obtain $\mathcal{G}_3$ of polynomial size. □

For the the technical details of the $\mathcal{G} \to \mathcal{G}_1 \to \mathcal{G}_2 \to \mathcal{G}_3$ constructions please see [41]. Figure 2 shows how these transformations may look like.

### 6 The Main Results

In this section, we prove the main results of the paper, namely that almost-sure energy parity stochastic games can be decided in $\mathsf{NP}$ and $\mathsf{coNP}$. The proofs are straightforward and follow from the much more involved characterization of almost sure energy parity objective in terms of the $\mathsf{Bailout}$ and $\mathsf{Gain}$ objectives established in Section 3 and their computational complexity analysis in Sections 4 and 5, respectively.

**Theorem 19.** *Given an SSG, energy level* $k^*$*, checking if a state* $s$ *is almost-sure winning for* $\mathsf{EN}(k^*) \cap \mathsf{PAR}$ *is in* $\mathsf{NP} \cap \mathsf{coNP}$.

*Proof.* Recall that we can compute the set $W$ from Definition 4 by iterating

$$W_i \stackrel{\text{def}}{=} \mathsf{AS}\left(\mathsf{Gain} \cap \mathbb{G}W_{i-1}\right) \cap \bigcup_k \mathsf{AS}\left(k\text{-}\mathsf{Bailout} \cap \mathbb{G}W_{i-1}\right)$$

starting with $W_0 \stackrel{\text{def}}{=} V$, until we reach the greatest fixed point $W$. Note that at step $i$ we need to solve almost sure $\mathsf{Gain}$ and almost sure $\bigcup_k \mathsf{AS}\,(k\text{-}\mathsf{Bailout})$, where the states of the game are restricted to $W_{i-1}$. There can be at most $|V|$ steps, because at least one state is removed in each iteration.

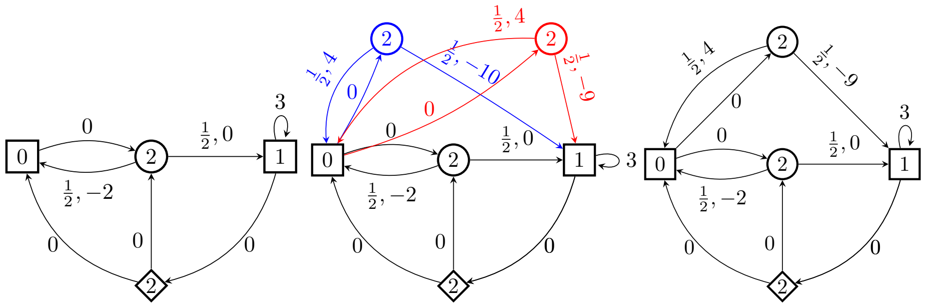

(a) The original game $\mathcal{G} = \mathcal{G}_1$ (b) The game $\mathcal{G}_2$ (c) The game $\mathcal{G}_3$

Fig. 2: An example game $\mathcal{G}$ (left) and the derived games. The strategy that always loops in the right-most state of $\mathcal{G}$ ensures a mean-payoff of 3. As this is the only MD strategy for maximizer that ensures a positive mean-payoff, a factor $f = 1$ is sufficient here and we have $\mathcal{G}_1 = \mathcal{G}$. In the derived game $\mathcal{G}_2$ in Fig. 2b there are as many trade-in options for the random state as there are MD minimizer's strategies in $\mathcal{G}_1$ (just two in this example). The blue one (top left) corresponds to minimizer going left and the red one (top right) to going up in $\mathcal{G}_1$. Maximizer almost-surely wins Gain in $\mathcal{G}$ iff he almost-surely wins a storage-parity condition (see Theorem 21) in $\mathcal{G}_3$.

It then suffices to check AS ($k$-Bailout $\cap\, \mathbb{G}W$) (i.e., AS ($k$-Bailout) for the subgame that consists only of the states of the fixed point $W$ for $k = k^*$. Note that this step can be skipped if $k^* \geq K$, the bound from Theorem 14.

Before we discuss how to use NP and coNP procedures to construct these sets and to conduct the final test on the fixed point $W$, we note that the '$\cap \mathbb{G}W_{i-1}$' does not add anything substantial, as these are simply the same tests and procedures conducted on the subgame that only consist of the states of $W_{i-1}$.

To obtain an NP procedure for constructing AS (Gain)—or, as remarked above, AS (Gain $\cap\, \mathbb{G}W_{i-1}$)—we can guess and validate its membership for each state $s$ *in* this set, using the NP result from Theorem 18, and we can guess and validate its non-membership for each state $s$ *not in* this set in NP, using the coNP result from Theorem 17. Similarly, we can guess and validate both the membership and the non-membership in $\bigcup_k$ AS ($k$-Bailout $\cap\, \mathbb{G}W_{i-1}$)—and of $\bigcup_k$ AS ($k$-Bailout $\cap\, \mathbb{G}W_{i-1}$) by analysing the subgame with only the states in $W_{i-1}$—by using the NP and coNP result, respectively, from Theorem 14.

Once we can construct these sets, we can also intersect them and check if a fixed point has been reached. (One can, of course, stop when $s \notin W_i$.)

We can now conduct the final check in NP using Theorem 18.

A coNP algorithm that constructs $W$ can be designed analogously: once $W_{i-1}$ is known, membership and non-membership of a state $s$ in AS (Gain $\cap\, \mathbb{G}W_{i-1}$) can be guessed and validated in coNP by Theorem 17 and by Theorem 18, respectively; and membership or non-membership of a state in $\bigcup_k$ AS ($k$-Bailout $\cap\, \mathbb{G}W_{i-1}$) can be guessed and validated in $\mathsf{coNP}$ using the $\mathsf{coNP}$ and $\mathsf{NP}$ part, respectively, of Theorem 14.

Once $W$ is constructed, we can conduct the final check in $\mathsf{coNP}$ using Theorem 17. □

This result, together with the upper bound on the energy needed to win energy-parity objective, allows us to solve the "unknown initial energy problem" [7], which is to compute the minimal initial energy level required.

**Corollary 20.** *For any state $s$, checking if there is $k$ such that* $\mathsf{AS}\,(\mathsf{EN}(k) \cap \mathsf{PAR})$ *holds is in* $\mathsf{NP} \cap \mathsf{coNP}$. *Also, for a given $k^*$, checking if $k^*$ is the minimal energy level required to win almost surely is in* $\mathsf{NP} \cap \mathsf{coNP}$ *as well.*

*Proof.* Due to Theorem 14, if there is an energy level $k$ for which $\mathsf{AS}\,(\mathsf{EN}(k) \cap \mathsf{PAR})$ holds, then it also holds for the bound $K$ whose size is polynomial in the size of the game. We can then simply calculate $K$ and then use $\mathsf{NP}$ and $\mathsf{coNP}$ algorithms from Theorem 19 for $\mathsf{AS}\,(\mathsf{EN}(K) \cap \mathsf{PAR})$.

As for the second claim, note that checking whether maximizer cannot win almost surely $\mathsf{EN}(k) \cap \mathsf{PAR}$ is also in $\mathsf{NP}$ and $\mathsf{coNP}$ as a complement of a $\mathsf{coNP}$ and an $\mathsf{NP}$ set, respectively. Therefore, for an $\mathsf{NP}/\mathsf{coNP}$ upper bound it suffices to simultaneously guess certificates for almost surely $\mathsf{EN}(k^*) \cap \mathsf{PAR}$ and not almost surely $\mathsf{EN}(k^* - 1) \cap \mathsf{PAR}$ and verify them in polynomial time. □

Finally, let us mention that the slightly more restrictive *storage-parity* objectives can also be solved in $\mathsf{NP} \cap \mathsf{coNP}$. These are almost identical to energy-parity except that, in addition, there must exist some bound $l \in \mathbb{N}$ such that the energy level never drops by more than $l$ during a run. This extra condition ensures that, if the storage-parity objective holds almost-surely, then there must exist a *finite-memory* winning strategy for maximizer.

**Theorem 21.** *One can check in* $\mathsf{NP}, \mathsf{coNP}$ *and pseudo-polynomial time if, for a given SSG* $\mathcal{H} \overset{def}{=} (V, E, \lambda)$, $k \in \mathbb{N}$ *and control state* $s \in V$, *maximizer can almost-surely satisfy* $\mathsf{ST}(k) \cap \mathsf{PAR}$ *from $s$.*

*Moreover, there is a bound $L \in \mathbb{N}$, polynomial in the number of states and the largest absolute transition reward, so that* $\mathsf{ST}(k) \cap \mathsf{PAR} = \mathsf{ST}(k, L) \cap \mathsf{PAR}$.

*Proof.* (sketch) This result follows by a simple adaptation of the proofs showing the same computational complexity of the $\mathsf{Bailout}$ objective (Section 4). See [41] for further details. □

*Example 22.* In the game in Fig. 1, maximizer cannot ensure the storage-parity condition $\mathsf{ST}(k) \cap \mathsf{PAR}$ for any initial energy level $k$. This is because it would imply the existence of a finite-memory almost-surely winning strategy, which as we have already argued, cannot be true. More intuitively, to prevent an intermediate energy drop by $l$ units, a winning maximizer strategy for storage-parity would need to stop moving left after observing the negative cycle in the leftmost state $l$ successive times. However, when maximizer moves right, this gives minimizer the chance to visit the rightmost bad state (with dominating odd priority 1). The chance of that happening is $(1/3)^l > 0$. In particular, this probability is $> 0$ for any value of the intermediate energy drop $l$. Therefore, for any fixed $l$, maximizer would need to move right infinitely often to satisfy storage and lose (against an optimal minimizer strategy that moves to the rightmost state).

### 7 Conclusion and Outlook

We showed that several almost-sure problems for combined energy-parity objectives in simple stochastic games are in $\mathsf{NP} \cap \mathsf{coNP}$. No pseudo-polynomial algorithm is known (just like for stochastic mean-payoff parity games [20]). All these problems subsume (stochastic) parity games, by setting all rewards to 0. Thus the existence of a pseudo-polynomial algorithm would imply that (stochastic and non-stochastic) parity games are in P, which is a long-standing open problem.

It is known that maximizer already needs infinite memory to win almost-surely a combined energy-parity objective in MDPs [40]. Our results do not imply anything about the memory requirement for optimal minimizer strategies in SSGs for this objective. We conjecture that memoryless minimizer strategies suffice. If this conjecture holds (and is proven), this would greatly simplify the $\mathsf{coNP}$ upper bound that we established for this problem.

A natural question is whether results on mean-payoff/energy/parity games can be generalized to a setting with multi-dimensional payoffs. Non-stochastic multi-mean-payoff and multi-energy games have been studied in [48,36,1]. To the best of our knowledge, the techniques used there, e.g. upper bounds on the necessary energy levels as in [36], do not generalize to stochastic games (or MDPs).

Multiple mean-payoff objectives in MDPs have been studied in [10,24], but the corresponding multi-energy (resp. multi-energy-parity) objective has extra difficulties due to the 0-boundary condition on the energy. I.e., even on Markov chains, and without any parity condition, it subsumes problems about multi-dimensional random walks. Some partial results on Markov chains and MDPs have been obtained in [13,2,3], but the decidability of the almost-sure problem for stochastic multi-energy-parity games (and MDPs) remains open.

### Acknowledgments

The work of all the authors was supported in part by EPSRC grant EP/M027287/1. Sven Schewe and Dominik Wojtczak were also supported by EPSRC grant EP/P020909/1.
